# T1-relaxation times along the corticospinal tract as a diagnostic marker in patients with amyotrophic lateral sclerosis

**DOI:** 10.3389/fnimg.2025.1549727

**Published:** 2025-02-13

**Authors:** Fiona Dierksen, Johanna S. Geibel, Janika Albrecht, Sabine Hofer, Peter Dechent, Amelie C. Hesse, Jens Frahm, Mathias Bähr, Jan C. Koch, Jan Liman, Ilko L. Maier

**Affiliations:** ^1^Department of Neurology, University Medical Center Göttingen, Göttingen, Germany; ^2^Department of Cognitive Neurology, University Medical Center Göttingen, Göttingen, Germany; ^3^Institute for Diagnostic and Interventional Radiology, University Medical Center Göttingen, Göttingen, Germany; ^4^Biomedical NMR, Max Planck Institute for Multidisciplinary Sciences, Göttingen, Germany; ^5^Department of Neurology, Klinikum Nürnberg, Nürnberg, Germany

**Keywords:** T1-mapping, T1 relaxometry, T1-relaxation time, amyotrophic lateral sclerosis, real-time MRI, corticospinal tract

## Abstract

**Background and purpose:**

In the differential diagnostic workup of amyotrophic lateral sclerosis (ALS), magnetic resonance imaging (MRI) is primarily used to rule out significant differential diagnoses. So far, whole-brain T1-mapping has not been assessed as a diagnostic tool in this patient population.

**Methods:**

We investigated the diagnostic potential of a novel T1-mapping method based on real-time MRI with 0.5 mm in-plane resolution and 4s acquisition time per slice. The study included patients aged 18 to 90 years who met the revised El Escorial criteria for at least possible ALS. T1-relaxation times were measured along the corticospinal tract in predefined regions of interest.

**Results:**

Twenty-nine ALS-patients and 43 control group patients (CG) were included in the study. Median ALS Functional Rating Scale revised (ALSFRS-R) was 37 (IQR, 35–44) points and the mean duration from symptom onset to MRI was 21 ± 17 (SD) months. ALS patients showed significantly higher T1-relaxation times in all ROIs compared to CG with mean differences in the hand knob of 50 ms (*p* < 0.001), corona radiata 24 ms (*p* = 0.034), internal capsule 27 ms (*p* = 0.002) and midbrain peduncles 48 ms (*p* < 0.001). There was a consistent negative correlation between the ALSFRS-R and T1-relaxation times in all ROIs.

**Conclusions:**

T1-relaxation times along the corticospinal tract are significantly elevated in ALS patients compared to CG and associated with lower ALSFRS-R. These results imply the analysis of T1-relaxation times as a promising diagnostic tool that can distinguish ALS patients from the control group. Ongoing longitudinal studies may provide deeper insights into disease progression and the effects of therapeutic interventions.

## 1 Introduction

Amyotrophic lateral sclerosis (ALS) is the most prevalent motor neuron disease, affecting up to 1 in 400 individuals over a lifetime (Wang and Patani, 2020). This neurodegenerative disorder is characterized by the progressive loss of both upper and lower motor neurons in the brain and spinal cord, leading to muscle weakness, paralysis, and ultimately death in a mean survival time of 3–5 years after symptom onset (Grad et al., 2017; Alhindi et al., 2022). Up to 30% of patients exhibit cognitive impairments, particularly in executive functions (Neary et al., 2000; Massman et al., 1996). The spectrum of cognitive deficits in ALS ranges from subtle changes to full frontotemporal dementia (van den Bos et al., 2019). Early detection is crucial to establish therapy as soon as possible (Abati et al., 2020), yet diagnostic latency ranges from 10 to 16 months (Richards et al., 2020), with a misdiagnosis rate between 20 and 42% (Kwan and Vullaganti, 2022; Belsh and Schiffman, 1996). This high error rate is partly due to the fact that diagnosis still relies on the existence of certain clinical symptoms while there is a lack of specific biomarkers (Sturmey and Malaspina, 2022). The currently available disease-modifying pharmacological treatments comprise riluzole and edaravone (only approved in certain countries) with modest benefits and newer gene therapies (Belsh and Schiffman, 1996), which can only be applied for patients with specific genetic mutations. Therapy should be started early to achieve most benefits (Hulisz, 2018).

Despite extensive research, the exact etiology of ALS remains unclear, with both genetic (Hulisz, 2018) and environmental (Schram et al., 2020; Korner et al., 2019) factors playing a role. Autopsy studies reveal motor cortex nerve fiber atrophy, particularly in the precentral gyrus (Cosottini et al., 2012). Microscopic examination shows significant motor neuron loss and reactive gliosis, marked by astrocyte and microglia proliferation in the brain and spinal cord (Vargas and Johnson, 2010; Saberi et al., 2015). Intracellular protein aggregates positive for the RNA-binding protein TDP-43 can be found in over 95% of all ALS cases (the exceptions are mainly patients with a SOD1-mutation) and are thus considered the pathological hallmark of the disease (Koski et al., 2021).

In clinical practice, imaging methods like magnetic resonance imaging (MRI) are mainly used to exclude other disorders that might clinically resemble ALS. MRI is so far not established as a specific diagnostic tool for the diagnosis of ALS, mainly because imaging findings are very heterogenous among ALS patients and often only appear later during disease course (Gupta et al., 2014). Key MRI findings in ALS include a hyperintensity on T2-weighted imaging along the corticospinal tracts (Simon et al., 2014), a loss of signal of the precentral gyrus on susceptibility-weighted imaging (SWI), known as the “motor band sign,” a superficial siderosis in the central sulcus on SWI as well as a spinal cord and corpus callosum atrophy (Bravo-Hernandez et al., 2020). Diffusion tensor imaging (DTI), measuring fractional anisotropy at the brainstem level, is so far the most effective parameter for distinguishing ALS patients from control group individuals (Maj et al., 2022). However, this imaging technique is limited by a low signal-to-noise ratio with prolonged imaging times as well as limited availability (Behler et al., 2023). All before mentioned MRI findings lack specificity and are prone to operator dependency. In addition, the MR-based findings cannot be used as early imaging markers for ALS, highlighting the need for more specific and quantitative imaging methods to enhance diagnostic accuracy.

T1-mapping represents a quantitative MRI technique that measures absolute T1-relaxation times of tissues and can be used to indicate microstructural changes in pathophysiological, disease specific processes. It emerged as a promising tool for detecting subtle changes in the brain and spinal cord (Vrenken et al., 2006; Shah et al., 2003; Steen et al., 2004), for example the water content in different brain tissues (Jiang et al., 2017), axonal membrane (Wainger et al., 2021) and myelin damages (Wang et al., 2011). By providing quantitative information about tissue properties, T1-mapping offers insights into disease progresses that may not be apparent on commonly used MRI such as T1- and T2-weighted MRIs, fluid attenuated inversion recovery (FLAIR) and hemoglobin sensitive T2^*^ or susceptibility weighted imaging (SWI) sequences (Shah et al., 2003). While it has been effectively utilized in various domains, including cardiovascular diseases (Kim et al., 2017; Radunski et al., 2014; Mewton et al., 2011), its potential for advancing neuroimaging in neurological disorders continues to grow. In particular for patients with suspected ALS commonly used MRI sequences are majorly used for differentiating other differential diagnoses without showing disease specific changes.

Aim of this study is to investigate if T1-relaxation times along the corticospinal tract differ between ALS and control patients free from CNS-disease and could serve as a disease specific marker.

## 2 Methods

### 2.1 Study design

We conducted an observational, experimental, monocentric and cross-sectional imaging study at the University Medical Center Göttingen (UMG), Germany, to investigate the diagnostic potential of a novel, real time T1-mapping MRI technique in ALS. The study included patients aged 18 to 90 years who met the revised El Escorial criteria for at least possible ALS (Brooks, 1994). Patients were recruited from the UMG outpatient department for motor neuron diseases. Total scan time was 22:16 min with 62 sequential single images per inversion-recovery experiment. All patients were required to provide informed consent prior to study inclusion and to tolerate the MRI procedure without significant movement during the measurement period. Patients who were unable to undergo MRI as well as patients with severe respiratory insufficiency or psychological distress, were excluded. This study was approved by the ethics committee of the UMG (4/3/19).

### 2.2 Patients

The study was conducted on three patient cohorts: spinal ALS (sALS), bulbar ALS (bALS), and a control group (CG). Spinal ALS was defined by the initial onset and clinical predominance of motor symptoms, such as muscle weakness and atrophy in the limbs and trunk, while bulbar ALS was defined as dysphagia and dysarthria as initial and predominant symptoms (Aiello et al., 2023; Sala et al., 2019; Hardiman et al., 2017). Patients were included in the control group for the study if they had no clinical- and MRI signs for neurodegenerative- (including generalized or focal brain atrophy exceeding the normal age range), neuroinflammatory- (including autoimmune-), ischemic- (including small vessel disease exceeding the normal age range) or hemorrhagic CNS disease as well as no signs for intracranial mass lesions. All CG and ALS patients underwent the same comprehensive clinical and neurology history and examinations, which included whole-brain imaging with T1-weighted, T2-weighted, FLAIR, DWI, as well as T2-weighted cervical spine imaging. T1-mapping of the whole brain and spinal cord in axial sections as well as the corpus callosum imaging in midsagittal sections was performed. Functional assessments included the finger tapping test, walking distance test, and timed speech test. Additionally, serum neurofilament levels (pNfH) were measured once at the start of the study for all participants. These groups were compared based on epidemiological and sociodemographic characteristics and the severity of neurological deficits was assessed using the ALSFRS-R (Cedarbaum et al., 1999) and the Edinburgh Cognitive and Behavioral ALS Screen (ECAS; Abrahams et al., 2014). Fast vs. slow progressors were determined by calculating the ALSFRS-R slope for each patient. The ALSFRS-R slope quantifies the rate of disease progression in ALS patients by calculating the monthly decline in the ALSFRS-R. It is determined as (48-ALSFRS-R score at assessment)/months from symptom onset to assessment.

### 2.3 Imaging

**Figure 1:**
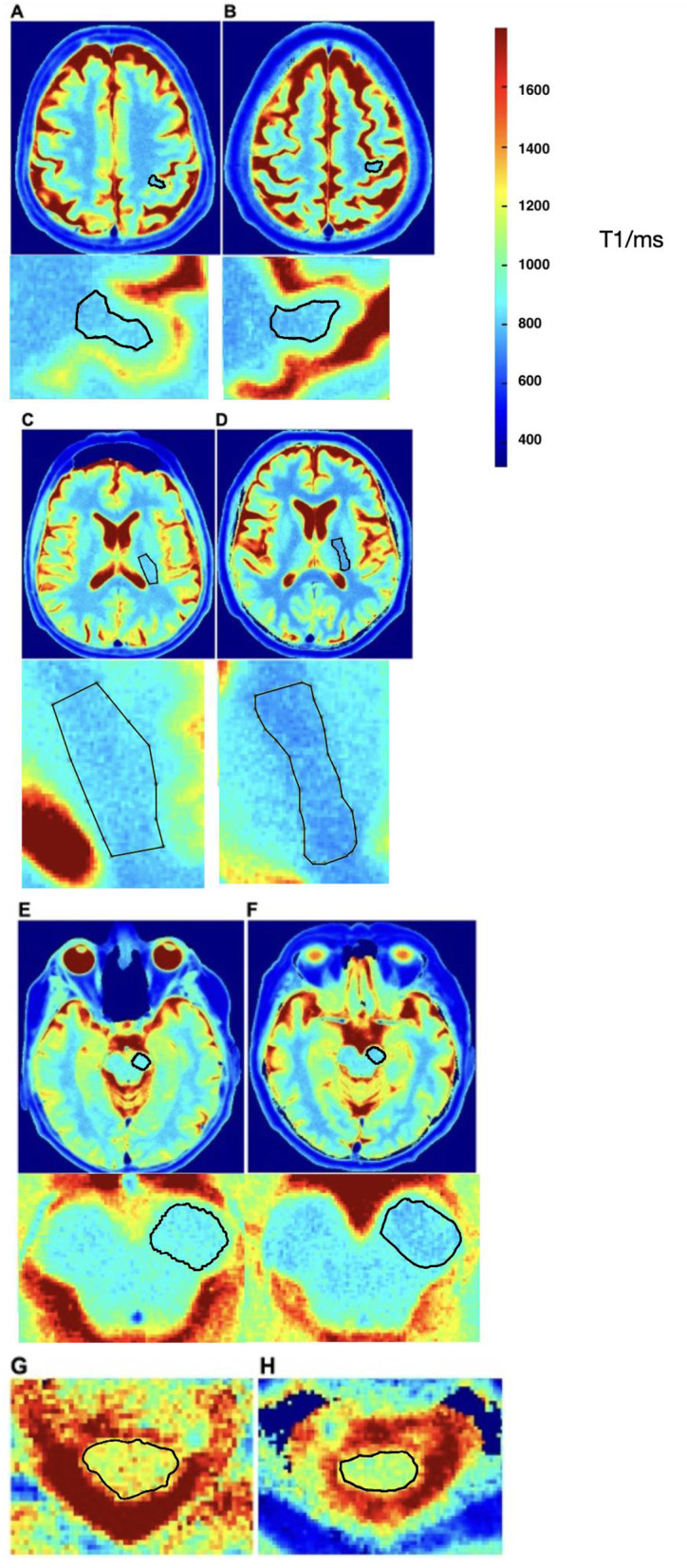
T1 maps of a representative ALS patient (left image series) and control group patient (right image series). T1-relaxation times are compared in multiple regions: the motor hand area of the left precentral gyrus **(A)** 963 ms vs. **(B)** 838 ms, posterior limb of the left internal capsule **(C)** 826 ms vs. **(D)** 755 ms, left crus cerebri **(E)** 961 ms vs. **(F)** 859 ms, and cervical spinal cord **(G)** 874 ms vs. **(H)** 810 ms. This figure demonstrates that T1-relaxation times are higher in ALS patients compared to the control group.

We investigated the diagnostic potential of a single-shot T1-mapping method of the whole brain utilizing a real-time MRI technique with an in-plane resolution of 0.5 mm and an acquisition time of 4 s per slice across our three patient cohorts. The imaging protocol consisted of standard T1-weighted and T2-weighted anatomical imaging (3D T1-weighted Turbo fast low angle shot (FLASH), acquisition time (TA): 8:26 min, T2-weighted Turbo-Spin-Echo, TA: 5:50 min) as well as of T1-mapping in transversal (TA: 4:37 min) and sagittal (TA: 3:09 min) orientation. Based on the transversal dataset, T1-relaxation times were assessed along the cerebral corticospinal tract within predefined regions of interest (ROIs) within the hand knob, corona radiata, internal capsule, and midbrain peduncles and are manually drawn in the color maps based on anatomical landmarks. The corpus callosum is used for the study because callosal fibers connect motor areas, allowing for an overview of motor fibers without the interference of crossing fiber pathways. Representative T1-maps of a CG and a patient with sALS are shown in Figure 1, with ROIs manually drawn along the underlying anatomical structures using the freehand selection tool in ImageJ/FIJI on color-coded T1 maps (Schindelin et al., 2012). In addition, based on the sagittal dataset, T1-relaxation times were assessed along the different regions of the corpus callosum in a sagittal plane (genu, anterior midbody, somatomotor, and splenium) as described before (Hofer et al., 2015). As this study aims to find disease- specific alterations of the T1-relaxation times of the corticospinal tract in patients with ALS, we also measured T1-relaxation times in a predefined ROI of the white matter of the occipital lobe. For the analysis, an index has been calculated by dividing the T1-relaxation times in the ROIs of the corticospinal tract by the T1-relaxation times of the occipital lobe white matter. This method is intended to correct for age related, physiological degeneration of white matter, as ALS majorly affects the corticospinal tract (Als et al., 2004). By doing so, we can assess the relative degeneration of the corticospinal tract compared to age-related degeneration of the deep white matter, providing an intra-individual correction.

### 2.4 Statistical analysis

Baseline characteristics of the three patient cohorts are reported as follows: continuous variables as mean ± standard deviation, ordinal variables as median (interquartile range), and nominal variables as percentages. Cohort comparisons were conducted using ANOVA for continuous variables, the Mann-Whitney rank sum test for ordinal variables (non-normally distributed), and the Chi-square test for binary variables, with significance set at *p* ≤ 0.05. R version 5.12.10 was used for all calculations.

Differences in T1-relaxation times between patients with sALS and bALS and CG were determined using univariate ANOVA. Logistic regression analysis was performed to investigate the predictive value of the T1-relaxation times for the diagnosis ALS compared to CG, including age as a confounding factor. Area under the receiver operator curve (AUROC) and Youden index were calculated for the hand knobs and midbrain peduncles for the endpoint ALS as these regions exhibited the most significant differences. AUROC vary from 0.5 for a model that correctly predicts outcome no better than chance to 1.0 for a model that perfectly discriminates between endpoints. Cut-off scores were defined as scores with maximal Youden-Index. Sensitivity and specificity are calculated by varying the threshold of the relaxation time and determining the true positive and true negative rates at each threshold. These values are then used to construct the ROC curve. Furthermore, clinical scores such as ALSFRS-R and the ALS specific ECAS score were correlated with T1-relaxation using Spearman correlations. Additionally, a *t*-test was conducted to assess the association between the right and left hand knob, as well as peduncles with sex, ALSFRS-R score and the ALSFRS-R slope score. A chi-square test was performed to compare ALS patients with CG regarding commonly used MRI markers, including atrophy of the corpus callosum and spinal cord, as well as T2 hyper- and hypointensities in the corticospinal tract, specifically in the subcortical region of the precentral gyrus. All *p*-values were not corrected for multiple testing due to the hypothesis-driven selection of regions of interest and the limited sample size, a strict correction could increase the risk of overlooking meaningful effects.

## 3 Results

We enrolled a total of 72 participants, encompassing 23 with sALS, 6 with bALS and 43 CG (Table 1). ALS typically concerns patients at peak with age of 74, the ALS incidence increases with each decade, especially after age of 40. There was no significant difference in age (sALS: 60 ± 12.1 vs. bALS: 59.7 ± 7.8 vs. CG: 55.7 ± 16.4; *p* = 0.492) between groups, while there was a higher percentage of male patients in the sALS group (18, 78.3%) compared to the bALS group (2, 33.3%) and CG (23, 53.5%, *p* = 0.057). Of the sALS patients, most had a probable laboratory supported (9, 39.1%) and probable (8, 34.8%) diagnosis, while all bALS patients had a probable and definite diagnosis (3, 50%, respectively). Mean time from symptom onset in the sALS group was 21.4 ± 17.2 months and 18.5 ± 11.3 in the bALS group (*p* = 0.701). In most cases the duration from diagnosis to study entry was < 12 months (sALS: 6 ± 5.63 vs. bALS: 8.0 ± 9.3, *p* = 0.506). A notable difference was observed in the ALSFRS-R, which was significantly higher in bulbar patients [sALS: 37 (35–44) vs. bALS: 39 (33–41), *p* < 0.001]. No statistical significance was observed for the ALS specific ECAS score between the two groups [sALS: 90 (81–93) vs. bALS: 89 (87–91), *p* = 0.483]. In addition, the vital capacity was significantly higher in the bALS group compared to the sALS group (sALS: 0.87 ± 0.12 vs. bALS: 0.7 ± 0.3, *p* = 0.044). Similarly, the maximum walking distance in 6 min was significantly higher in the bALS group (sALS: 306 ± 194 vs. 522 ± 301, *p* = 0.040).

**Table 1:** Baseline characteristics of patients with spinal and bulbar amyotrophic lateral sclerosis and control group.

	**Spinal ALS (*n* = 23)**	**Bulbar ALS (*n* = 6)**	**Control group (*n* = 43)**	*p*-value^*^
Age (mean ± SD)	60.0 ± 12.1	59.7 ± 7.8	55.7 ± 16.4 (*n* = 42)	0.491
Sex male (*n*, %)	18 (78.3)	2 (33.3)	23 (53.5)	0.057
BMI (mean ± SD)	26.42 ± 4.2	25.0 ± 2.1	27.1 ± 4.1 (*n* = 7)	0.634
Revised El Escorial criteria:				0.143
possible (*n*, %)	4 (17.4)	0.00		
probable laboratory supported (*n*, %)	9 (39.1)	0.00		
probable (*n*, %)	8 (34.8)	3 (50)		
definite (*n*, %)	2 (8.7)	3 (50)		
Symptom onset to inclusion in the study (mean month ± SD)	21.4 ± 17.2	18.5 ± 11.3		0.701
Diagnosis to inclusion in the study (mean month ± SD)	6 ± 5.63	8.0 ± 9.3		0.506
Vitalcapacity (mean liter ± SD)	0.87 ± 0.12	0.7 ± 0.3		0.044
Muscle Strength MRC Scale 0–200 (mean ± SD)	171 ± 171	180 ± 180		0.099
Serum Neurofilaments (NfH) [pg/ml] (mean ± SD)	2703.3 ± 3065.1	4705 ± 4656.6		0.365
ALSFRS-R 0–48 (median, IQR)	37 (35–43.5)	39 (33–40.5)		< 0.001
ECAS ALS specific (median, IQR)	90 (81–93)	88.5 (86.5–90.5)		0.483
ECAS (median, IQR)	120.5 (107–126.25)	127.5 (118.25–130)		0.152
Time up and go (mean ± SD)	11.6 ± 6.5	9.9 ± 4.2		0.555
Max. Walking distance in 6 min (mean ± SD)	306.1 ± 193.7	522 ± 301.4		0.040

ALS, Amyotrophic Lateral Sclerosis; SD, standard deviation; BMI, body mass index; IQR, inter quartile range.

^*^Comparisons of all means between all 3 groups were performed by ANOVA, comparisons of means between 2 groups (spinal and bulbar ALS) were performed using *t*-tests, categorial variables were compared using cross-tables und using the Chi^2^ test.

### 3.1 T1-relaxation times in patients with sALS, bALS and CG

Overall, T1-relaxation times were higher in all predefined ROIs along the corticospinal tract in ALS patients compared to CG (Table 2). These differences were consistently statistically significant for the sALS vs. the CG groups. The highest differences of the T1-relaxation times have been found between the sALS and CG group for both hand knobs (right 49.9 ms, left 42 ms; *p* < 0.01) and both midbrain peduncles (right 47.5 ms, left 41.7 ms; *p* < 0.001). The analysis of the corpus callosum revealed significantly higher T1-relaxation times in the genu for both sALS and bALS patients vs. CG with a mean difference of >100 ms in both ALS groups (*p* = 0.001). In contrast, T1-relaxation times in the somatomotor region and anterior midbody were lower in ALS patients compared to CG (*p* < 0.01). There were no significant differences for T1-relaxation times in the splenium of the corpus callosum between all groups (*p* > 0.2). Supplementary Figure 1 shows the difference of relaxation times in every ROI between the ALS patients and the CG group.

**Table 2:** T1-relaxation times in predefined white matter regions of interest along the corticospinal tract and corpus callosum in patients with spinal and bulbar amyotrophic lateral sclerosis and control group.

	**sALS (*****n*** = **23)**		**bALS (*****n*** = **6)**		**CG (*****n*** = **43)**		**Mean difference sALS vs. CG**	**Mean difference bALS vs. CG**	**Mean difference sALS vs. bALS**	**sALS vs. CG**	**bALS vs. CG**	**sALS vs. bALS**
	**Mean ms**	**SD**	**Mean ms**	**SD**	**Mean ms**	**SD**	**ms**	**ms**	**ms**		***p*** **-value** ^*^	
Hand knob right	888.11	48.12	884.29	49.88	838.20	43.78	49.91	46.09	3.83	< 0.001	0.023	0.865
Hand knob left	874.82	52.89	869.82	34.45	832.81	45.63	42.01	37.01	5.00	0.002	0.064	0.829
Corona radiata right	795.99	42.45	781.23	25.86	772.32	41.74	23.67	8.91	14.76	0.034	0.615	0.427
Corona radiata left	797.61	49.14	779.11	25.07	771.10	41.39	26.51	8.01	18.51	0.024	0.648	0.384
Internal capsule right	815.24	32.46	800.54	24.45	788.48	32.40	26.77	12.07	14.70	0.002	0.387	0.312
Internal capsule left	815.42	28.79	789.71	24.59	783.97	29.83	31.45	5.73	25.71	< 0.001	0.656	0.056
Midbrain peduncle right	929.39	33.41	916.29	22.39	881.90	39.21	47.50	34.39	13.11	< 0.001	0.042	0.374
Midbrain peduncle left	925.94	31.63	914.10	11.82	884.25	39.18	41.69	29.85	11.84	< 0.001	0.072	0.381
Corpus callosum genu	970.57	104.86	932.17	49.49	830.38	40.24	140.19	101.79	38.40	0.001	0.001	0.396
Corpus callosum anterior midbody	809.91	46.10	790.33	21.78	886.75	20.72	−76.84	−96.42	19.58	< 0.001	< 0.001	0.326
Corpus callosum somatomotor	786.86	39.87	781.59	34.62	894.63	68.27	−107.77	−113.03	5.26	< 0.001	0.003	0.770
Corpus callosum splenium	784.10	41.14	791.86	37.69	773.50	23.22	10.60	18.36	−7.77	0.498	0.281	0.679
Index hand knob right	1.13	0.06	1.13	0.05	1.09	0.05	0.04	0.04	< 0.01	0.009	0.048	0.908
Index hand knob left	1.12	0.05	1.10	0.05	1.08	0.04	0.04	0.02	0.02	0.004	0.282	0.503
Index corona radiata right	1.01	0.04	1.00	0.04	1.01	0.04	< 0.01	−0.01	0.01	0.878	0.453	0.473
Index corona radiata left	1.02	0.03	0.99	0.05	1.01	0.03	0.01	−0.02	0.03	0.266	0.136	0.044
Index capsula int. post. Right	1.04	0.04	1.03	0.04	1.03	0.04	0.01	−0.01	0.01	0.620	0.693	0.467
Index capsula int. post. Left	1.04	0.03	1.00	0.04	1.02	0.04	0.02	−0.02	0.04	0.062	0.180	0.005
Index midbrain peduncle right	1.18	0.04	1.18	0.07	1.15	0.06	0.03	0.02	0.01	0.038	0.410	0.726
Index midbrain peduncle left	1.18	0.05	1.16	0.06	1.15	0.06	0.03	< 0.01	0.03	0.030	0.863	0.293

ALS, Amyotrophic Lateral Sclerosis; SD, standard deviation; int. post., interna posterior; sALS, spinal ALS; bALS, bulbar ALS; CG, Control group.

^*^Comparisons of mean T1-relaxation times have been performed using ANOVA between all 3 groups.

The analysis of the adjusted T1-relaxation times of the corticospinal tract (index of T1-relaxation times of the ROIs of the corticospinal tract divided by the white matter T1-relaxation times in the occipital lobe) revealed significantly higher indices for the hand knob and midbrain peduncles in the sALS group vs. the CG group (*p* < 0.05). In the bALS group, only a significantly higher index was found for right hand knob (1.13 ± 0.05 vs. 1.09 ± 0.05, *p* = 0.048).

**Figure 2:**
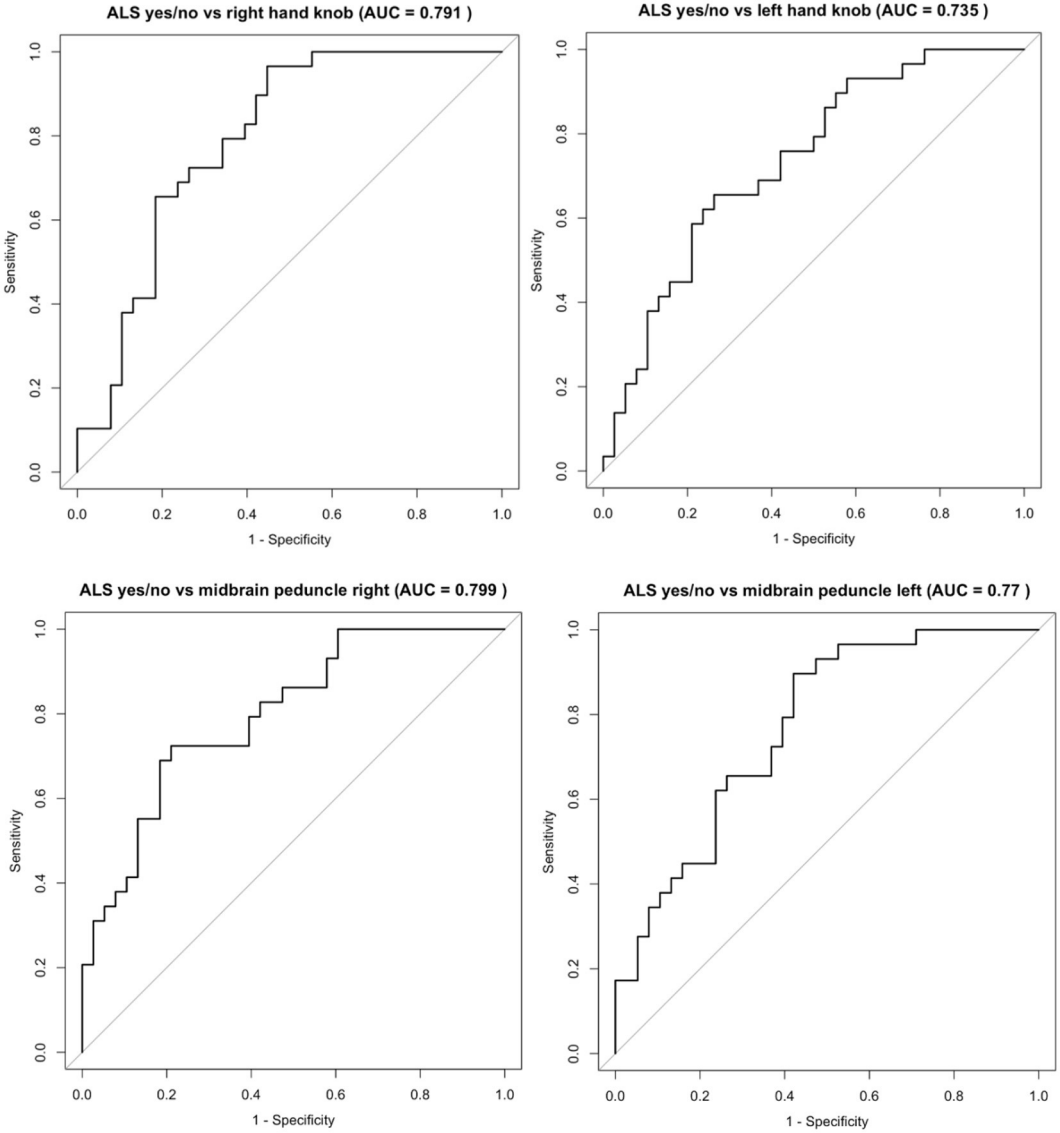
ROC Analysis of ALS diagnosis vs. right and left hand knob, midbrain peduncle right and left.

The ROC curve analysis demonstrated that all anatomical regions showed *p*-values below 0.001, indicating statistically significant differences and acceptable discrimination (Figure 2). The right hand knob demonstrated a moderate predictive value, with an AUC of 0.791, a Youden index of 0.518, and a T1-relaxation time cut-off of 835.3 ms. Similarly, the left hand knob showed a moderate AUC of 0.735, a Youden index of 0.392, and a cut-off of 856.7 ms. The right midbrain peduncle also exhibited moderate predictive performance, with an AUC of 0.799, a Youden index of 0.514, and a cut-off of 908.2 ms. The left midbrain peduncle achieved an AUC of 0.770, a Youden index of 0.475, and a cut-off of 897.1 ms for T1-relaxation time (Figure 2).

### 3.2 Predictive value of T1-relaxation times for ALS diagnosis

When combining T1-relaxation times and patient age into a multivariable logistic regression model, T1-relaxation times remained a significant independent predictor for the ALS diagnosis (Figure 2). For the diagnosis sALS, T1-relaxation times of the hand knobs (OR 1.04, CI 1.02–1.06, *p* < 0.001), internal capsules (OR 1.03, CI 1.01–1.06, *p* = 0.004), the midbrain peduncles (OR 1.04, CI 1.02–1.06, *p* < 0.001), corpus callosum genu (OR 1.04, CI 1.02–1.08, *p* = 0.012), corpus callosum anterior midbody (OR 0.94, CI 0.85–0.97, *p* = 0.027) and the corpus callosum somatomotor region (OR 0.93, CI 0.85–0.97, *p* = 0.015) were significant predictors (Figure 2).

### 3.3 Correlation of T1-relaxation times and ALSFRS-R

There was a consistent negative correlation between T1-relaxation times of the corticospinal tract and the ALSFRS-R (Table 3), which was statistically significant for the right hand knob (*r* = −0.400, *p* = 0.032), the right internal capsule (*r* = −0.370, *p* = 0.048) and the left midbrain peduncle (*r* = −0.381, *p* = 0.041). For the corpus callosum we found a strong trend toward a positive correlation in the anterior midbody (*r* = 0.366, *p* = 0.051) and the somatomotor region (*r* = 0.318, *p* = 0.093) in contrast to the other ROIs.

**Table 3:** Correlation between amyotrophic lateral sclerosis functional rating scale (ALSFRS-R) and T1-relaxation times in predefined white matter regions of interest in patients with ALS and control group.

**T1-relaxation times (ms)/ALSFRS-R**	**Correlation coefficient**	*p*-value^*^
Hand knob right	−0.400	0.032
Hand knob left	−0.325	0.085
Corona radiata right	−0.196	0.308
Corona radiata left	−0.278	0.144
Internal capsule right	−0.370	0.048
Internal capsule left	−0.423	0.022
Midbrain peduncle right	−0.351	0.062
Midbrain peduncle left	−0.381	0.041
Corpus callosum genu	−0.666	< 0.001
Corpus callosum anterior midbody	0.366	0.051
Corpus callosum somatomotor	0.318	0.093
Corpus callosum splenium	−0.304	0.108
Index hand knob right	0.086	0.663
Index hand knob left	−0.103	0.601
Index corona radiata right	0.156	0.427
Index corona radiata left	−0.183	0.350
Index capsula int. post. right	0.042	0.833
Index capsula int. post. left	−0.237	0.224
Index midbrain peduncle right	0.132	0.503
Index midbrain peduncle left	−0.270	0.164

ALSFRS-R, amyotrophic lateral sclerosis functional rating score.

^*^Spearman correlation between ALSFRS-R and T1-relaxation time.

The strongest negative correlations were observed for the right hand knob (*r* = −0.400, *p* = 0.032), left internal capsule (*r* = −0.423, *p* = 0.022) and genu of the corpus callosum (*r* = −0.666, *p* < 0.001). Other regions, including the corona radiata or the corpus callosum anterior midbody, somatomotor region or splenium were not statistically significant. The correlation between T1-relaxation times and the ECAS score specific to ALS showed no significant correlations (Supplementary Table 2).

### 3.4 T1-relaxation times in clinical subgroups

The T1-relaxation times of the hand knobs showed no significant differences (Supplementary Table 3) between male and female patients (right hand knob: 880.4 ± 34.9 vs. 884.4 ± 43.7, *p* = 0.825; left hand knob: 870.2 ± 43.3 vs. 863.2 ± 29, *p* = 0.626). However, in the left peduncle, male patients exhibited significantly higher T1-relaxation times compared to female patients (left peduncle: 929.4 ± 30 vs. 906 ± 19.6, *p* = 0.025). When comparing fast and slow ALS progressors based on the ALSFRS-R-Slope score, no significant differences in the T1-relaxation times were observed in either the right and left hand knob or the right and left peduncle (Right hand knob: fast progressors: 870.5 ± 38.9 vs. slow progressors: 890.9 ± 31.6, *p* = 0.161). Similarly, no significant differences in T1-relaxation times were detected when comparing patients with low vs. high ALSFRS-R scores (Right hand knob: low ALSFRS-R: 872.9 ± 20.4 vs. high ALSFRS-R: 891.4 ± 47.4, *p* = 0.229). They were defined based on the median ALSFRS-R score at the time of examination, with patients scoring below the median classified as low and those above the median classified as high.

### 3.5 Signs for ALS in commonly used MRI

In ALS patients, 6.8% (*n* = 5) showed T2 hyperintensity in the corticospinal tract, with no cases of T2-hypointensity of the motor cortex (“low signal rim”) or spinal cord atrophy on commonly used MRI imaging. The prevalence of corpus callosum atrophy was not different between ALS patients and controls (*p* = 0.747).

## 4 Discussion

Our study demonstrated that mean T1-relaxation times along the corticospinal tract were significantly higher in ALS patients compared to CG. In particular, this was true for the hand knobs and the midbrain peduncles, where significant differences persisted using an index with T1-relaxation times of the occipital lobe, which is less likely to be involved in the ALS specific disease process. This indicates that T1-relaxation times along the corticospinal tract can be used as a quantitative marker for disease activity in ALS, while commonly used MRI imaging is often unremarkable. Additionally, higher T1-relaxation times were associated with lower ALSFRS-R scores in multiple regions along the corticospinal tract, indicating a direct link between T1-relaxation times and functional patient status.

ALS is characterized by the rapid progressive degeneration of upper and lower motor neurons, leading to paralysis of striated muscles over time. The El Escorial criteria, which are used for the clinical diagnosis of ALS, require clinical signs of damage to both upper and lower motor neurons in at least two body regions, which can often only be detected in the later stages of the disease (de Jongh et al., 2022). For affected patients, diagnostic uncertainty and delayed diagnosis significantly impair quality of life and cause psychological distress. Moreover, this delay postpones the initiation of effective supportive and pharmacological therapies, which can prolong survival and improve quality of life (Miller et al., 2009). This uncertainty of diagnosis can also be due to a heterogeneous onset of symptoms as well as the overlap of other neurological disorders (Kwan and Vullaganti, 2022; Belsh and Schiffman, 1996). In this respect, the use of T1-mapping to qualify changes in T1-relaxation times along the corticospinal tract might be an additional tool for an early diagnosis of ALS and also to serve as an imaging method to distinguish between other differential diagnosis in the advanced stage of the disease, e.g., in severe peripheral neuropathies where central nervous structures are unaffected. The mean time from diagnosis to imaging in our study was < 1 year, which supports the hypothesis that T1-relaxation times might serve as an early imaging marker of the disease.

Multiple MRI techniques have been studied extensively for the diagnosis of ALS, however, only few is used in clinical practice as technical requirements and expertise are majorly found in specialized centers. These advanced imaging techniques are highly effective non-invasive tools that can be utilized to investigate and measure structural, functional, and metabolic abnormalities (Ilieva et al., 2023; Menke et al., 2017). In contrast, commonly used MRI (T1- or T2-weighted imaging, FLAIR and T2^*^ sequences) scans of the brain and spinal cord usually reveal abnormalities in the later stages of the disease and are challenging to interpret on an individual basis, despite studies on large patient cohorts showing typical atrophy patterns (van der Burgh et al., 2019; Wimmer et al., 2020). As a result, MRI currently in many centers is primarily used to rule out differential diagnoses (Ludolph et al., 2021). Newer imaging tools exist, such as the “Neurite Orientation Dispersion and Density Imaging (NODDI)” and the “quantitative Magnetization Transfer Imaging (qMTI),” which are used to detect pathophysiological processes such as loss of inhibitory interneurons and progressive gliosis in multiple cerebral fiber pathways alongside other MRI- and PET-based techniques (Barritt et al., 2018; Broad et al., 2019). However, these experimental imaging methods are limited by small sample sizes and inadequate clinical characterization of included patients, thereby restricting their application in clinical practice for diagnosis and prognosis assessment (Chi et al., 2014). Additional disadvantages of these imaging techniques include prolonged acquisition times, which often cannot be tolerated by ALS patients being investigated in supine position.

T1-mapping has been used in multiple neurological diseases to detect and quantify disease-specific changes in neuronal tissues, especially in diseases where imaging changes are only subtle or prone to misdiagnosis (Vrenken et al., 2006; Shah et al., 2003; Steen et al., 2004; Maier et al., 2022). These changes have been found especially in the white matter, where they were related to microstructural features such as myelin and non-myelin water content, axonal size and axonal density as well as iron content (Hofer et al., 2015; Gelman et al., 2001). In a study investigating whole brain T1-mapping in patients with idiopathic normal pressure hydrocephalus (iNPH), increased periventricular T1-relaxation times have been identified (Maier et al., 2022). In iNPH, these regions have been shown to include areas with marked microangiopathy and ischemia, leading to demyelination and axonal loss. Axonal loss, in particular, is a hallmark of ALS due to its effect on motor neurons and may explain the increased T1-relaxation times observed in patients compared to CG. This hypothesis is supported by the finding of hyperintensities along the corticospinal tract, which can be detected with commonly used MRI in more advanced ALS stages. The latter has also been found in cerebral small vessel disease, which also include demyelination and axonal damage as a microstructural hallmark. Another reason why the increased T1-relaxation times along the corticospinal tract in ALS patients might correspond to the degree of axonal loss is the negative correlation to the ALSFRS-R, which quantifies motor function, which again decreases with the degree of axonal loss. Why there were no significant correlations between the T1-relaxation times and the ECAS remains speculative. We mainly focused the measurements of the T1-relaxation times in motor fiber tracts and not regions for higher cognitive functions and, therefore, the degeneration of these fiber tracts may not correlate to the degree of cognitive impairment.

The reason why we mainly observed significant differences in sALS to CG patients, but not less in bALS to CG patients might be because bALS patients were underrepresented in our study. However, bulbar neurons are mainly involved in the disease process in bALS, which also could explain the lack of significant differences in mean T1-relaxation times along the corticospinal tract in this patient group compared to CG. The latter explanation, however, contradicts the observation, that we could not show differences in T1-relaxation times between sALS and bALS patients. This might indicate that axonal loss in the corticospinal tract is also occurring in bALS patients, but to a less pronounced extent.

We observed a distinct pattern of changes of T1-relaxation times along the corpus callosum with increased T1-relaxation times in the genu and decreased T1-relaxation times in the anterior midbody and somatomotor region, while there were no differences in the splenium. The latter is in line with DTI studies of the corpus callosum identifying predominant changes of the fractional anisotropy in the genu, anterior midbody and somatomotor region (Cardenas et al., 2017; Münch et al., 2022). The exact explanation, why in the anterior midbody and somatomotor region of the corpus callosum T1-relaxation times—in contrast to the genu and corticospinal tract—show a decrease and not an increase in our study, remains speculative. One explanation could be the difference in microstructure within these regions with small axon sizes and less degree of myelination in the genu compared to the anterior midbody and somatomotor region of the corpus callosum, where axons are larger in diameter and degree of myelination is much higher (Aboitiz et al., 1992). These microstructural, anatomical differences in these regions also have been shown to translate in significant differences in T1-relaxation times in control group subjects (Hofer et al., 2015).

It has been shown that white matter T1-relaxation times are increasing physiologically in the white matter with age (Dinçer et al., 2023). By including age in our multivariable logistic regression model, we excluded its influence on the results and could show that increased T1-relaxation times along the corticospinal tract remain an independent predictor for ALS diagnosis regardless of age.

Our study has several limitations. First, there are several factors which can influence T1-relaxation times in the white matter that we could not correct for. These factors include lifestyle factors such as smoking, alcohol consumption and sleep duration, which all have been shown to influence T1-relaxation times (Wang et al., 2015; Topiwala et al., 2017; Grumbach et al., 2020). All these factors, including cerebral microangiopathy, are very likely to affect all white matter regions of the brain. Therefore, we tried to overcome these various confounders by analyzing an index of corticospinal tract ROIs and a standardized ROI in the occipital lobe, which revealed a persistent increase in T1-relaxation times of the hand knobs and midbrain peduncles in sALS patients. Another key limitation of our study is the variability in ROI localization and size across patients, which may introduce inconsistencies in the measured T1-relaxation times of the hand knob, corona radiata and internal capsule and due to differences in anatomical alignment and rater-dependent factors. However, as is commonly observed in cerebral white matter diseases caused by microangiopathy, white matter changes can be patchy and are unevenly distributed. Regarding bALS, the number of patients included in this study is low and therefore this study cannot answer the question, if this subgroup of patients can be discriminated from sALS patients and CGs. Furthermore, our study is limited by being monocentric and non-blinded, with very small sample sizes. The ALS group is highly heterogeneous, and we did not standardize the time point after symptom onset at which patients were included. There was also no spinal imaging performed, which limits our ability to capture the full spectrum of ALS-related pathology. Lastly, the small number of bALS patients included makes it difficult to determine whether this subgroup can be clearly differentiated from sALS patients and control group (CG). This limitation is also why we did not perform *p*-value correction, emphasizing that the results should be interpreted as showing marginal significance. Future studies with larger sample sizes are essential to validate these findings and confirm the observed trends.

In conclusion, the finding of consistently increased T1-relaxation times along well-defined and reproducible ROIs along the corticospinal tract represents a promising, quantifiable imaging biomarker for the pathophysiological processes associated with neurodegeneration in ALS. In ongoing research, we are focusing on longitudinal data to identify changes in T1-relaxation times over time and correlate these changes with different clinical courses as well as analyze anatomical patterns of disease progression. Future studies should continue to build on these insights to better understand disease dynamics.

## Data Availability

The raw data supporting the conclusions of this article will be made available by the authors, without undue reservation.
