# Supplementary material for: T1-relaxation times along the corticospinal tract as a diagnostic marker in patients with amyotrophic lateral sclerosis
Source: Front Neuroimaging. 2025 Feb 13;4:1549727. doi: 10.3389/fnimg.2025.1549727 (PMC11865248; doi:10.3389/fnimg.2025.1549727)

Supplementary Material

| Supplementary table 1: Predictive value of T1-relaxation times for the diagnosis of amyotrophic lateral sclerosis | | | | | | | | |
| --- | --- | --- | --- | --- | --- | --- | --- | --- |
|  |  |  | |  | |  |  |  |
|  | | | T1-relaxation times | | | | Age | |
| White matter region of interest | | | OR (95%CI) | | p-value | | OR (95%CI) | p-value |
| Hand knob right | | | 1.04 (1.02-1.06) | | < 0.001 | | 0.96 (0.91-1.0) | 0.082 |
| Hand knob left | | | 1.02 (1.01-1.04 | | 0.003 | | 0.98 (0.93-1.02) | 0.288 |
| Corona radiata right | | | 1.01 (0.99-1.02) | | 0.147 | | 1.0 (0.96-1.05) | 0.862 |
| Corona radiata left | | | 1.01 (0.99-1.02) | | 0.127 | | 1.0 (0.96-1.04) | 0.968 |
| Internal capsule right | | | 1.03 (1.0-1.05) | | 0.014 | | 0.99 (0.95-1.04) | 0.793 |
| Internal capsule left | | | 1.03 (1.01-1.06) | | 0.004 | | 0.99 (0.95-1.03) | 0.635 |
| Midbrain peduncle right | | | 1.04 (1.02-1.06) | | < 0.001 | | 1.0 (0.96-1.04) | 0.988 |
| Midbrain peduncle left | | | 1.04 (1.02-1.06) | | < 0.001 | | 1.02 (0.98-1.06) | 0.354 |
| Corpus Callosum Genu | | | 1.04 (1.02-1.08) | | 0.012 | | 0.99 (0.87-1.12) | 0.909 |
| Corpus Callosum anterior midbody | | | 0.94 (0.85-0.97) | | 0.027 | | 1.14 (0.98-1.5) | 0.186 |
| Corpus Callosum somatomotor | | | 0.93 (0.85-0.97) | | 0.015 | | 1.08 (0.95-1.3) | 0.311 |
| Corpus Callosum splenium | | | 1.01 (0.99-1.04) | | 0.373 | | 0.97 (0.88-1.06) | 0.522 |
| Index Hand knob right | | | 1.15 (1.04-1.30) | | 0.013 | | 1.0 (0.97-1.05) | 0.644 |
| Index Hand knob left | | | 1.19 (1.05-1.36) | | 0.008 | | 1.03 (0.98-1.06) | 0.203 |
| Index Corona radiata right | | | 0.97 (0.85-1.10) | | 0.621 | | 1.03 (0.99-1.07) | 0.143 |
| Index Corona radiata left | | | 1.03 (0.89-1.19) | | 0.712 | | 1.02 (0.99-1.06) | 0.168 |
| Index Capsula int. post. right | | | 1.02 (0.90-1.15) | | 0.784 | | 1.05 (0.99-1.06) | 0.162 |
| Index Capsula int. post. left | | | 1.13 (0.98-1.31) | | 0.091 | | 1.04 (1.0-1.08) | 0.056 |
| Index midbrain peduncle right | | | 1.12 (1.02-1.25) | | 0.023 | | 1.04 (0.99-1.08) | 0.069 |
| Index midbrain peduncle left | | | 1.19 (1.07-1.37) | | 0.004 | | 1.07 (1.02-1.13) | 0.007 |
| OR = Odds Ratio, CI = Confidence Interval  *Multivariate logistic regression was performed to determine if T1-relaxation times serve as an independent predictor of ALS, accounting for age as a confounding factor. | | | | | | | | |

| Supplementary table 2: Correlation between Edinburgh Cognitive and Behavioral ALS Screen (ECAS) and T1-relaxation times in predefined white matter regions of interest in patients with ALS. | | |
| --- | --- | --- |
| T1-relaxation times (ms) / ECAS | Correlation Coefficient | p-value* |
| Hand knob right | -0.188 | 0.415 |
| Hand knob left | -0.119 | 0.609 |
| Corona radiata right | 0.003 | 0.989 |
| Corona radiata left | -0.093 | 0.690 |
| Internal capsule right | -0.278 | 0.223 |
| Internal capsule left | 0.001 | 0.996 |
| Midbrain peduncle right | -0.258 | 0.259 |
| Midbrain peduncle left | -0.260 | 0.255 |
| Corpus Callosum Genu | -0.018 | 0.940 |
| Corpus Callosum anterior midbody | -0.247 | 0.280 |
| Corpus Callosum somatomotor | 0.195 | 0.396 |
| Corpus Callosum splenium | 0.086 | 0.711 |
| Index Hand knob right | -0.421 | 0.057 |
| Index Hand knob left | -0.113 | 0.625 |
| Index Corona radiata right | -0.330 | 0.145 |
| Index Corona radiata left | -0.309 | 0.172 |
| Index Capsula int. post. right | -0.382 | 0.087 |
| Index Capsula int. post. left | 0.037 | 0.875 |
| Index midbrain peduncle right | -0.390 | 0.081 |
| Index midbrain peduncle left | -0.020 | 0.932 |
| ECAS: Edinburgh Cognitive and Behavioral ALS Screen  *Spearman correlation between ECAS and T1-relaxation times | | |

| Supplementary table 3: Association of T1-relaxation times with clinical subgroups. | | | | | | | | | | | | |
| --- | --- | --- | --- | --- | --- | --- | --- | --- | --- | --- | --- | --- |
|  | Hand knob right | | | Hand knob left | |  | Peduncles right | |  | Peduncles left | |  |
|  | mean ± SD |  | p-value | mean ± SD |  | p-value | mean ± SD |  | p-value | mean ± SD |  | p-value |
| ALSFRSr | Low: 891.4 ± 47.4 | High: 872.9 ± 20.4 | 0.229 | Low: 873.6 ± 38.5 | High: 860.7 ± 37.1 | 0.395 | Low: 936.7 ± 35.3 | High: 913.7 ± 25.4 | 0.075 | Low: 930.7 ± 35.3 | High: 913.6 ± 20.7 | 0.157 |
| Sex | Males: 880.4 ± 34.85 | Females: 884.4 ± 43.7 | 0.825 | Males: 870.2 ± 43.3 | Females: 863.2 ± 29 | 0.626 | Males: 928.8 ± 33.6 | Females: 915.9 ± 22.4 | 0.252 | Males: 929.4 ± 30 | Females: 906 ± 19.6 | 0.025 |
| ALSFRS-Slope | Fast: 870.5 ± 38.9 | Slow: 890.9 ± 31.6 | 0.161 | Fast: 862.3 ± 43.9 | Slow: 870.3 ± 32.5 | 0.610 | Fast: 934.2 ± 37 | Slow: 915.8 ± 25.2 | 0.161 | Fast: 925.3.2 ± 28.1 | Slow: 918.2 ± 30.5 | 0.543 |
| SD = standard deviation, ALSFRSr = ALS Functional Rating Scale | | | | | | | | | | | | |
| * Comparison of ALS patients across four regions by applying t-tests: the right and left hand knobs and the right and left peduncles. Comparisons were performed based on three clinical variables: (1) ALSFRSr scores (low vs. high), (2) sex (male vs. female) and (3) ALSFRS-Slope, distinguishing between fast and slow disease progressors. | | | | | | | | | | | | |

Supplementary figure 1: Boxplot of T1-relaxation times of the cortiospinal tract by ROI region between ALS patients and the CG.


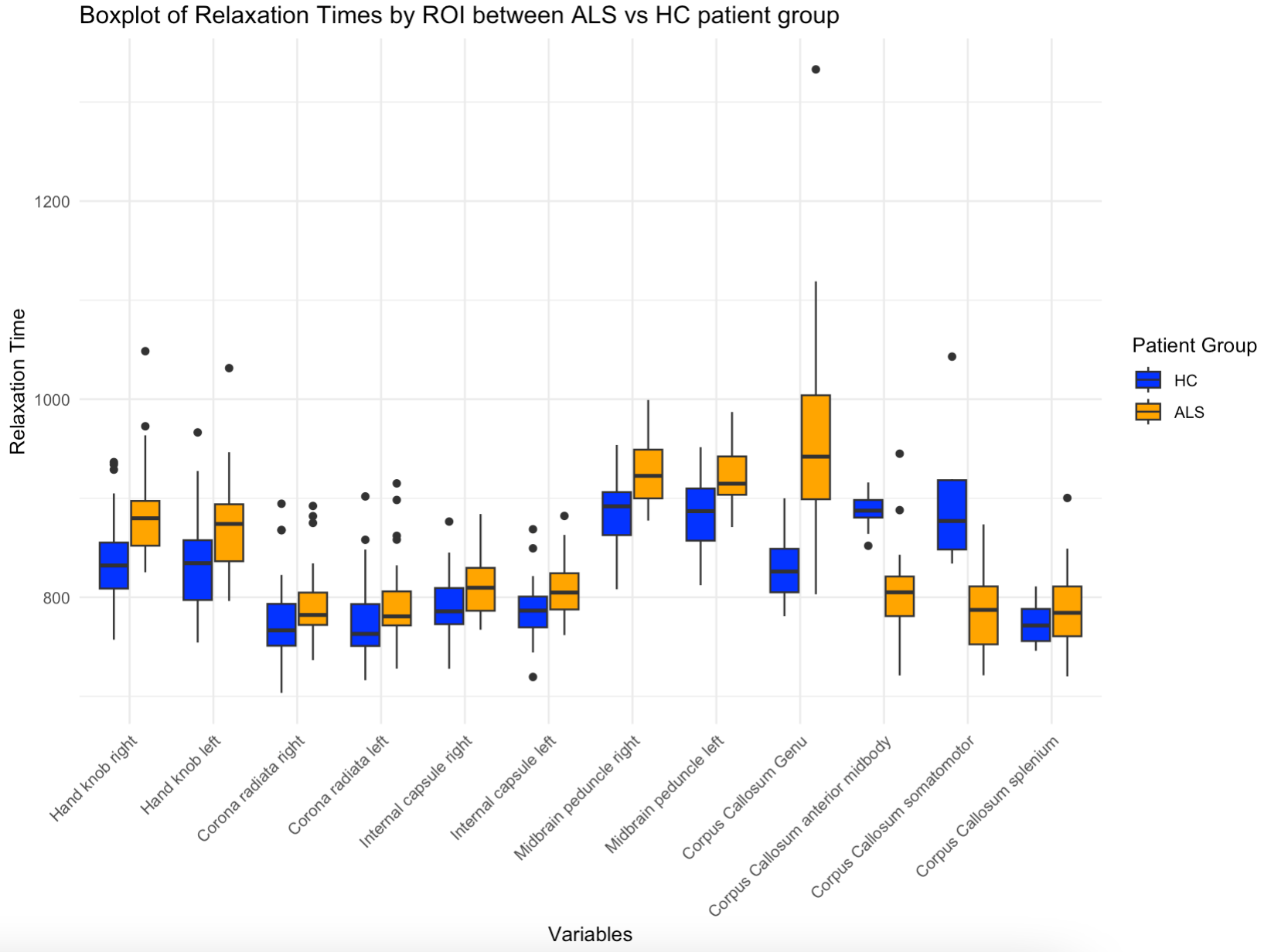

Supplement: Supplementary file 1 [file Table_1.docx]
